# Influence of Dry Soil on the Ability of Formosan Subterranean Termites, *Coptotermes formosanus*, to Locate Food Sources

**DOI:** 10.1673/031.011.16201

**Published:** 2011-11-23

**Authors:** Mary L. Cornelius, Weste L.A. Osbrink

**Affiliations:** United States Department of Agriculture, Agricultural Research Service, Southern Regional Research Center, 1100 Robert E. Lee Blvd., New Orleans, LA 70124, USA

**Keywords:** barrier, feeding, foraging, tunneling

## Abstract

The effect of barriers of dry soil on the ability of Formosan subterranean termites, *Coptotermes formosanus* Shiraki (Isoptera: Rhinotermitidae), to construct tunnels and find food was evaluated. Termite movement and wood consumption in a three—chambered apparatus were compared between treatments with dry soil in the center container and treatments where the soil in the center container was moist. When a wood block was located in the release container, termites fed significantly more on that block, regardless of treatment or soil type. In the treatment with dry clay, none of the termites tunneled through the dry clay barrier to reach the distal container. When termites had to tunnel through a barrier of dry sand, topsoil, or clay to reach the sole wood block, there was no effect on wood consumption for the sand treatment, but there was significantly less feeding on wood in the treatments with dry topsoil or clay. When foraging arenas had a section of dry sand in the center, the dry sand significantly reduced tunneling in the distal section after 3 days, but not after 10 days. There was a highly significant effect on the ability of termites to colonize food located in dry sand. Only one feeding station located in dry sand was colonized by termites, compared with 11 feeding stations located in moist sand.

## Introduction

Subterranean termites are highly susceptible to desiccation, making moisture a critical factor for survival. Many studies have examined the influence of moisture on the tunneling and feeding behavior of subterranean termites (Evans 2003; Su and Puche 2003; Arab and Costa-Leonardo 2005; Green et al. 2005; McManamy et al. 2008; Gautam and Henderson 2011a, 2011b). In order to tunnel into dry soils, termites need to relocate water molecules from moist soil into the dry soil by using their salivary reservoirs as water sacs (Grube and Rudolph 1999a, 1999b; Gallagher and Jones 2010). In a study where the only available food source was located on dry soil, mortality of the Formosan subterranean termite *Coptotermes formosanus* Shiraki (Isoptera: Rhinotermitidae) was high, even though termites were able to travel freely between moist sand and dry soil. Clusters of desiccated termite bodies were observed on the surface of the dry soil in many of the replicates, possibly due to rapid desiccation caused by contact with dry soil (Cornelius and Osbrink2010).

There is an interaction between moisture availability and soil type. Both moisture retention and availability are affected by particle size. Water retention is higher in soils with smaller particles sizes, but moisture availability is greater in soils with larger particles sizes. Hence, termites are able to obtain water from sandy soils with lower water content than clay soils (Lys and Leuthold 1994). Moisture availability affected *C. formosanus* preferences for different soil types. When soils were moist, termites were significantly more likely to aggregate in topsoil than in potting soil or peat moss. In moist soils, termites aggregated in the soil with the smallest particle size and the least organic matter. When soils were dry, termites were significantly more likely to move into the soils containing the largest amount of organic matter, peat moss and potting soil, than into the soils with the least amount of organic matter, sand and clay (Cornelius and Osbrink 2010).

The objective of this study was to examine how barriers of dry soil affect the ability of termites to construct tunnels and find new food sources. This study evaluated the effect of dry topsoil, sand, or Montmorrillonite clay barriers on the movement, survival, and wood consumption of Formosan subterranean termites *C. formosanus* Shiraki. This study also evaluated tunnel construction and the ability of termites to find food in foraging arenas with a barrier of dry sand compared with arenas without any dry sand.

## Materials and Methods

### Termite collection and maintenance

Termites were collected from three field colonies that were located in different areas (> 1000 m apart) of City Park, New Orleans, Louisiana, USA. Termites were collected by using cylindrical irrigation valve boxes (22.5 × 14.8 cm) (NDS, www.ndspro.com) buried in the ground so that the lid was level with the surface of the soil. The boxes were filled with blocks of wood (spruce, *Picea* sp.). The collected termites were maintained in the laboratory in 5.6 L covered plastic boxes containing moist sand and blocks of spruce (8 × 4 × 0.5 cm) until they were used in experiments. Termites were used in experiments within two months of collection.

### Soil types

For this study, three soil types were used: Play Sand silica sand (Quikrete, www.quikrete.com); a uniformly fine Montmorillonite clay (Ecological Laboratories, www.microbelift.com); and GardenPlus topsoil (Hope Agri Products). The particle size of the clay was < 0.002 mm. The particle size of the sand and topsoil was determined using sieve analysis. For sand, > 99% of particles ranged from 0.30–0.85 mm, and < 1% were very coarse particles of 1–2 mm. For topsoil, 78% of particles were ≤ 0.25 mm, 12% ranged from 0.26–0.85 mm, and 10% were > 0.85 mm. The composition of the topsoil was determined using a soil macronutrients kit (LaMotte Company, www.lamotte.com). A soil texture test determined that the topsoil was comprised of approximately 60% sand, 3% silt, and 37% clay. The macronutrient content was measured as nitrogen (11.4 kg/hectare), phosphorus (171 kg/hectare), and potassium (251 kg/hectare).

### Three-chambered testing apparatus

Three clear polystyrene cylindrical screwtop containers (9 cm high × 7 cm diameter) were connected using two 5 cm length pieces of PVC tubing (6.35 mm inner diameter × 11.11 mm outside diameter × 2.38 mm wall) (Nalgene, www.nalgene.com) inserted through holes in the sides of the containers and sealed in place with hot glue applied with a glue gun. In the two end containers, there was 50 g sand (Play Sand, Quikrete), moistened with 10 mL of distilled water to thoroughly moisten sand. The end containers were filled with sand to a height of 1 cm. The center container was filled with the substrate (topsoil, sand, or clay) to a height of 6 cm in order to make sure that termites had to tunnel through the substrate to find the tube leading to the distal container. In control replicates, the substrate in all three containers was moistened with distilled water. A soil moisture meter (Spectrum Technologies, www.specmeters.com) was used to establish moisture levels of 80% saturation for topsoil, sand, and clay. In treated replicates, the substrate in the center container was dry. Spruce (*Picea* sp.) blocks (4.2 × 3.8 × 1 cm) were oven-dried at 90 °C for 24 hours and weighed.

Two experiments were conducted to evaluate tunneling behavior of termites through a dry soil barrier, and to determine whether access to wood in the release container affected termite behavior. In the first experiment, a wood block was placed on top of the sand in both the release container and the distal container. In the second test, a wood block was placed on top of the sand in the distal container only so that there was no wood in the release container. A moistened 5.5 cm filter paper disk was placed on top of the sand in the release container to provide a limited food source to prevent starvation.

For each experiment, there were 12 replicates of each treatment with four replicates from each of three colonies for each treatment, except there were only six replicates of each treatment for the clay in the first experiment. Groups of 200 termites (190 workers, 10 soldiers) were placed in the release container. Each three-chambered testing apparatus was placed in a dark incubator (28 °C, 97% RH). After 30 days, the number of termites in each container was counted and all wood blocks were removed, cleaned, oven-dried at 90 °C for 24 hours, and weighed. Wood consumption was measured by determining the weight loss of blocks.

### Foraging arenas

The foraging arenas consisted of two plexiglass sheets (41 × 41 × 0.3 cm). Each bottom sheet had a border (2 cm length × 0.2 cm height). On each bottom sheet, a single large washer (3 cm diameter × 0.2 cm height) was placed in the center and eight small washers (0.8 cm diameter × 0.2 cm height) were arranged at a distance of 8 cm from the border and from other washers as spacers. On the top sheet, three 1 cm diameter holes were cut and covered with a small plastic screwtop container (5 cm diameter × 4.5 cm height) where the top of the lid was glued to the sheet such that the hole in the lid was aligned with the hole in the sheet. One container was located in the bottom corner of the foraging arena at a distance of 2 cm from the bottom and left edge of the sheet and served as the release site for termites. Termites were released by placing them in the container, covering them with a moist filter paper, and then turning the container over and attaching it to the lid. The filter paper prevented termites from spilling out when the container was turned over and also provided a food source. The other two containers served as feeding stations and were located in the center of the arena 8 cm apart, and at a distance of 22 cm from the bottom of the arena, 14 cm from the top of the arena, and 12–14 cm from each side. A small block (2 × 1.8 × 0.5 cm) of spruce was inserted into the hole in the top plexiglass sheet in each feeding site.

Foraging arenas were filled with sand in an area within the borders (37 × 37 cm). Sand was evenly distributed throughout the bottom sheet and thoroughly moistened with distilled water. There were two treatments and 12 replicates, with four replicates from each of three colonies for each treatment. In one treatment, all sand within the arena was moist. In the other treatment, a center section with dry sand was created by removing the sand from a center section (12 × 37 cm) and filling this area in with dry sand. The area tunneled by termites in each foraging arena was measured. Tunnels were traced on transparent film using a blue sharpie and were photographed. The tunneling area was measured using SigmaScan Pro 5.0 (SigmaScan 1999). The tunneling area was compared for three sections: the release section, the center section, and the distal section. The 1 cm length area on the release section along the border of the arena was excluded from analysis in order to compare three tunneling sections of equal size (12 × 37 cm). One feeding station was located in the center section and one feeding station was located in the release section. In the dry sand treatment, one feeding station was located in the dry section and one feeding station was located in the moist section. Tunneling area was compared after three days and 10 days for each section. Also, the number of feeding stations colonized and the time until discovery was compared after 20 days.

### Statistical analysis

In the two experiments with the three—chambered apparatus, the number of termites in release, center, or distal containers, weight loss of blocks in release or distal containers, and termite survival was compared for moist and dry treatments using either a *t*—est or a Mann—Whitney *U* rank sum test in cases where the tests for normality or equal variances failed. In the experiment with wood blocks located in both the release and distal containers, the combined weight loss of the two wood blocks in each replicate was compared for moist and dry treatments using either a *t*—est or Mann—Whitney *U* rank sum test.

The distribution of termites between the release, center, and distal containers within each treatment for each soil type was compared using a one—way ANOVA, except that a one—way Kruskal—Wallis ANOVA on Ranks was used if tests for normality or equal variances failed. Means were separated using Tukey's Honestly Significant Difference test.

In foraging arena tests, tunneling areas for the three sections after three days and 10 days were compared for the dry center treatment using a Kruskal—Wallis one—way ANOVA because the test for normality failed. Tunneling areas for the three sections after three days and 10 days in the moist center treatment were compared using a one-way ANOVA. Means were separated using Tukey's Honestly Significant Difference test for both experiments. The tunneling areas in each section for each time period were compared for the two treatments using a Mann—Whitney *U* rank sum test. The number of feeding stations colonized in the release section and the center section for each treatment was compared using a Wilcoxon Signed Rank test. All analyses were conducted using SigmaPlot 11.0 (Systat Software 2008).

## Results and Discussion

### Three-chambered testing apparatus

There were no significant differences in survival between treatments for any of the soil types for either experiment (Table 1). In the present study, termites needed to be able to construct tunnels in dry substrate in order to reach a container with moist sand and wood. Termites were able to move enough moisture to construct tunnels through a barrier of dry soil without suffering increased mortality. In a previous study, termites suffered significantly greater mortality when the only food source was located on dry substrate than when food was located on moist substrate for tests with topsoil, sand, and clay (Cornelius and Osbrink 2010). Gautam and Henderson (2011) also found significant mortality by *C. formosanus* in no—choice feeding tests on wood blocks with dry (0–3%) or low (22–24%) moisture content.

In Experiment 1, there were significant differences in the distribution of termites in both treatments for topsoil (moist center: H = 10.9, *p* < 0.01; dry center: *F* = 5.1, df = 2, 35, *p* < 0.01), sand (moist center: H = 6.1, *p* < 0.05; dry center: H = 7.3, *p* < 0.05), and clay (moist center: *F* = 5.2, df = 2, 17, *p* < 0.05; dry center: *F* = 552.8; df = 2, 17, *p* < 0.01).

There were significantly more termites in release containers among replicates with dry centers for topsoil and clay, but not for sand. There were no significant differences in wood consumption between the two treatments for either topsoil or sand. For clay, there was significantly more consumption of wood blocks in the distal container when the clay was moist than when it was dry. In the dry treatment, 85% of the termites were located in the release container and none of the termites were able to reach the distal container (Table 2).

When termites were released into a container with a block of wood, they fed significantly more on the block in the release container than in the distal container, regardless of treatment or soil type (Table 2). Termites are likely to feed more on the first block they encounter (Oi et al. 1996). Once termites initiate feeding on a block, secretions from the labial glands stimulate feeding behavior (Reinhard et al. 1997; Reinhard and Kaib 2001a; Casarin et al. 2003), and trail pheromones are used to recruit additional workers (Reinhard and Kaib 2001b).

In Experiment 2, there were significant differences in the distribution of termites in the treatments with a moist center for topsoil (H = 24.1, *p* < 0.01), sand (*F* = 3.7; df = 2, 35, *p* < 0.05), and clay (H = 18.4; *p* < 0.01). In treatments with a dry center, there were no significant differences in the distribution of termites for either topsoil (*F* = 2.7, df = 2, 35, *p* = 0.08) or sand (H = 3.6; *p* = 0.17). However, there were significantly more termites in the release container than the other two containers (H = 21.5, *p* < 0.01) for clay. There was significantly more wood consumption in moist treatments than dry treatments for topsoil and clay, but not for sand (Table 3).

**Figure 1.**
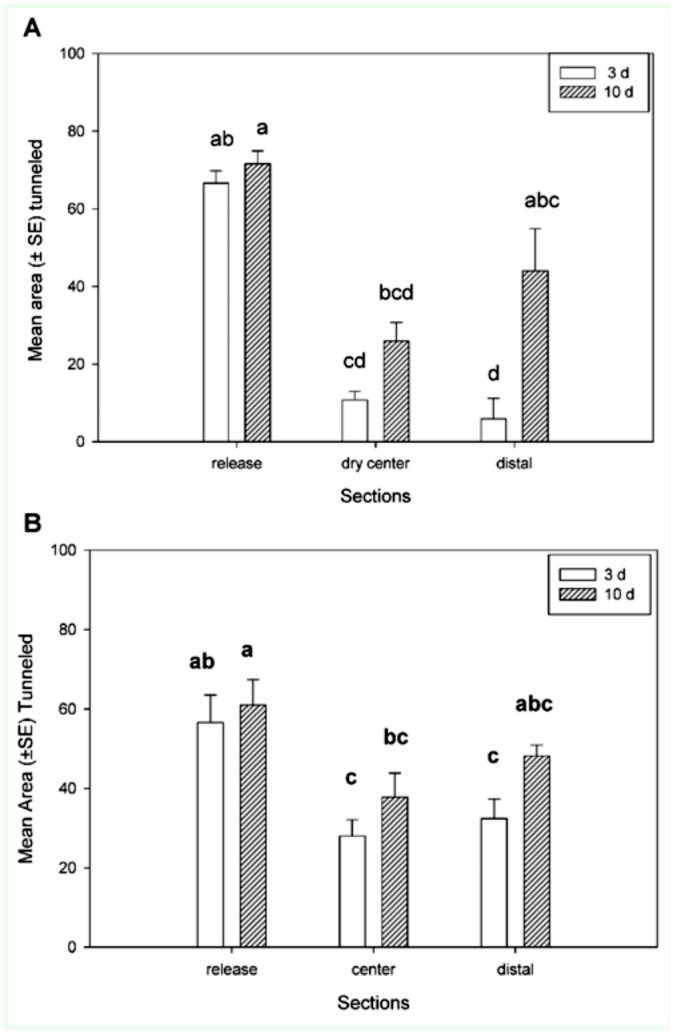
Mean area ±SE tunneled (cm^2^) by termites in foraging arenas divided into three sections: release, center, and distal. Tunneling areas measured after three days and 10 days. Treatment with (A) dry sand in center section and (B) with all moist sand. Bars followed by different letters were significantly different (Tukey's Honestly Significant Difference test: *p* < 0.05). High quality figures are available online.

The ability of termites to tunnel through dry soil was affected by particle size. Termites were able to tunnel through the dry sand and colonize the block in the distal container in both experiments. In Experiment 2, the barrier of dry topsoil significantly decreased wood consumption compared to replicates with moist topsoil. In both of the experiments, dry clay was a significant barrier to termite movement. Termites fed significantly less on the block located in the distal container, and the majority of termites were located in the release container after 30 days.

### Foraging arenas

**Figure 2.**
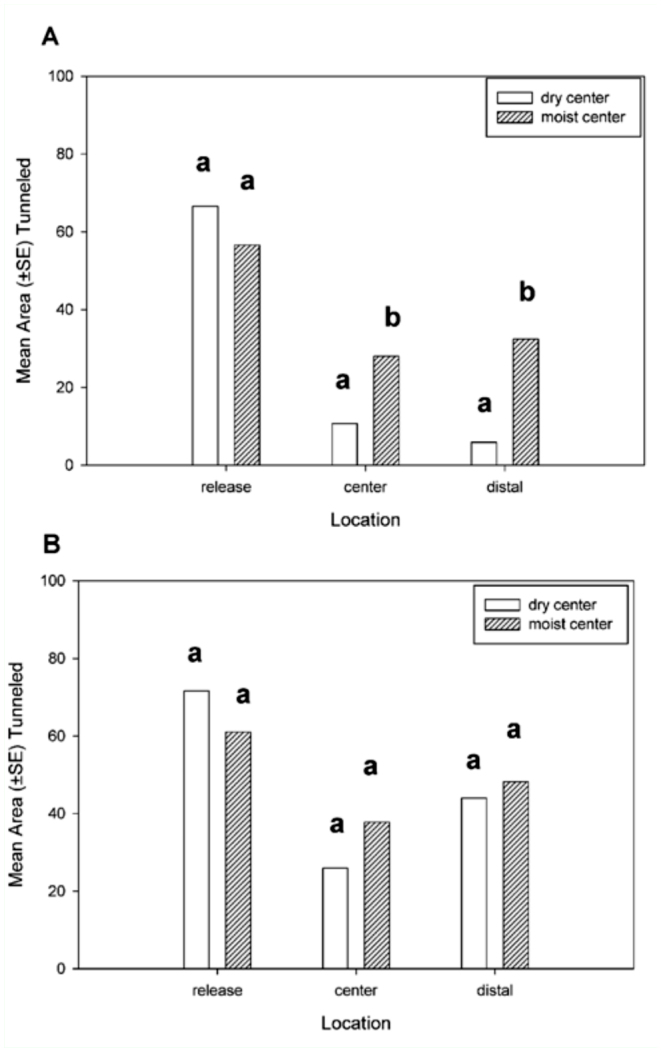
Mean area — SE tunneled (cm^2^) by termites in foraging arenas divided into three sections: release, center, and distal. Area tunneled after (A) three days and (B) 10 days. Bars followed by different letters for each section were significantly different between the two treatments (Mann-Whitney *U*: *p* < 0.05). High quality figures are available online.

There were significant differences in tunneling areas in the three sections in the dry center treatment (H = 49.5, *p* < 0.01) and the moist center treatment *(F*= 6.2, df = 5, 71, *p* < 0.01). In both treatments, the tunneling area was significantly greater in the release section than in the center and distal sections after three days, but not after 10 days. After 10 days, tunneling in the release section was significantly greater than tunneling in the center, but not the distal section for both treatments (Figure 1A, 1B). In a comparison of the tunneling areas in each section after three days, there was no difference in the area tunneled in the release section between treatments (*p* = 0.29), but there was significantly more tunneling in the center (*p* < 0.01) and distal sections (*p* < 0.01) in the treatment with moist sand in the center (Figure 2A). In a comparison of the tunneling areas in each section after 10 days, there were no significant differences in any of the areas tunneled in any of the sections between the two treatments (Figure 2B).

**Figure 3.**
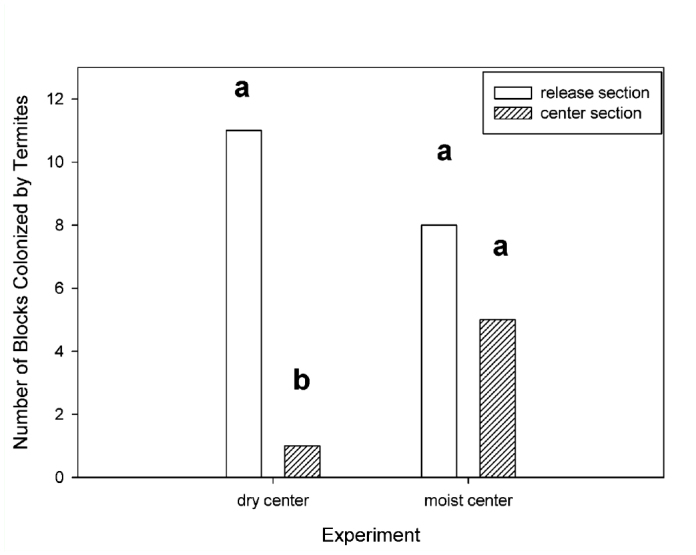
Number of blocks colonized by termites in feeding stations located in the release section and the center section in treatments with dry sand in the center section or with all moist sand. Bars followed by different letters for each treatment were significantly different (Wilcoxon Signed Rank test: *p* < 0.05). High quality figures are available online.

In the dry center treatment, the number of feeding stations colonized by termites in the release section was significantly greater than the number colonized in the center section (Wilcoxon Signed Rank: p < 0.01), but there was no significant difference in the number of feeding stations colonized in each section in the moist center treatment (Wilcoxon Signed Rank: *p* = 0.31) (Figure 3).

In foraging arenas, the dry center section initially created a barrier for termites that significantly reduced the area tunneled in the distal section compared with arenas with moist centers after three days, but not after 10 days. In both treatments, termites constructed significantly more tunnels in the release sections after three days, but not after 10 days. There was a highly significant effect of dry sand on the ability of termites to colonize feeding stations. Only one feeding station located in dry sand was colonized by termites compared with 11 feeding stations located in the release section. In the moist arenas, there was no significant difference between the number of feeding stations located in the release section and the center section colonized by termites.

### Conclusions

Several studies have demonstrated that subterranean termites preferentially tunnel in soil with a higher moisture content (Evans 2003; Su and Puche 2003; Arab and Costa-Leonardo 2005). Studies have also demonstrated that subterranean termites can relocate water to dry substrates (Grube and Rudolph 1999a, 1999b; Gallagher and Jones 2010). These results demonstrated that Formosan subterranean termites were able to tunnel through dry sand barriers more effectively than barriers of dry topsoil or clay, and that dry clay appeared to have the most impact on termite movement. However, termites were much less likely to colonize wood located on dry sand than moist sand. The cost of relocating sufficient water to maintain humid conditions when both the wood and the soil are dry appears to present a substantial obstacle to foraging termites.

**Table 1.**
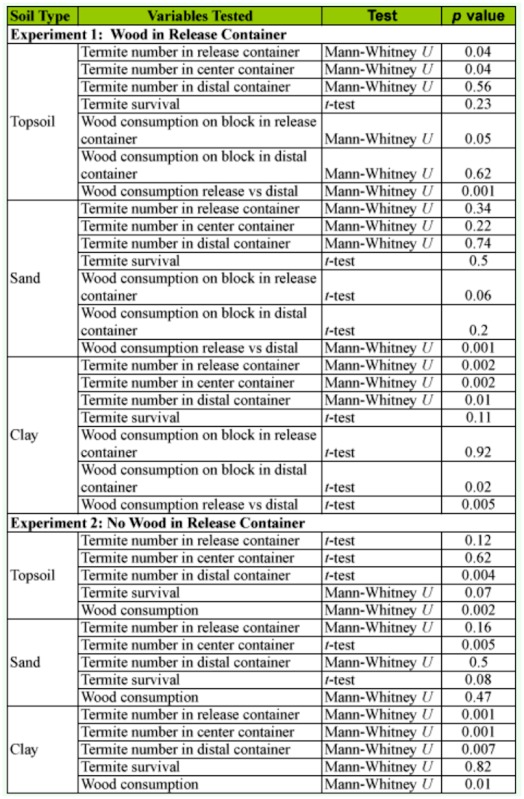
Comparison of number of termites in each location, percent survival and wood consumption between the moist and dry center container treatments.

**Table 2.**
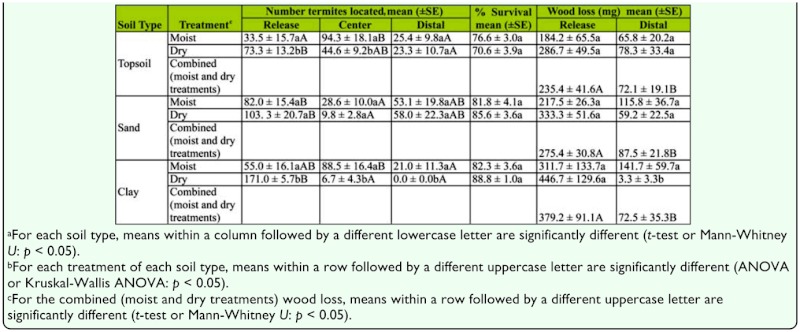
Experiment 1: Wood Block in release container.

**Table 3.**
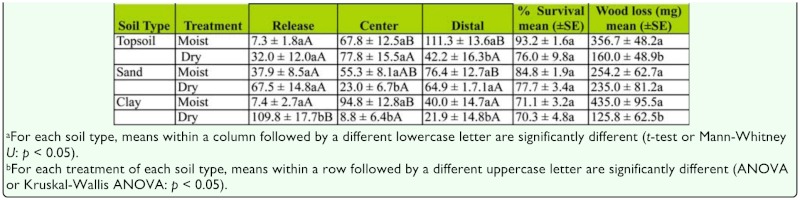
Experiment 2: No wood in release container.
